# (2*R*,4*S*,5*S*)-5-Meth­oxy-4-methyl-3-oxohept-6-en-2-yl benzoate

**DOI:** 10.1107/S2414314621009512

**Published:** 2021-09-17

**Authors:** Ann-Christin Schmidt, Lyuba Iovkova, Martin Hiersemann

**Affiliations:** a Fakultät für Chemie und Chemische Biologie, Technische Universität Dortmund, Organische Chemie, 44227 Dortmund, Germany; University of Aberdeen, Scotland

**Keywords:** crystal structure, methyl­ation, epimerization, structural elucidation

## Abstract

The title compound, C_16_H_20_O_4_, was synthesized in the course of the total synthesis of fusaequisin A in order to verify and confirm the configurations of the stereogenic centers and to exclude the possibility of epimerization during the methyl­ation process. The crystal structure of the title compound at 100 K has ortho­rhom­bic (*P*2_1_2_1_2_1_) symmetry. The absolute configuration was determined by anomalous dispersion and agrees with the configuration of the allylic alcohol used in the synthesis.

## Abstract 2

The title compound was synthesized in the course of the total synthesis of fusaequisin A in order to verify and confirm the configurations of the stereogenic centers and to exclude the possibility of epimerization during the methyl­ation process. The absolute configuration was determined by anomalous dispersion and agrees with the configuration of the allylic alcohol used in the synthesis.

## Graphical abstract

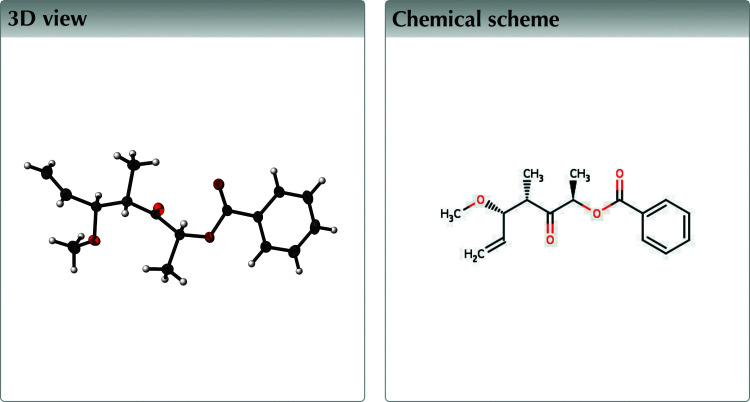

## Structure description

**Figure 1:**
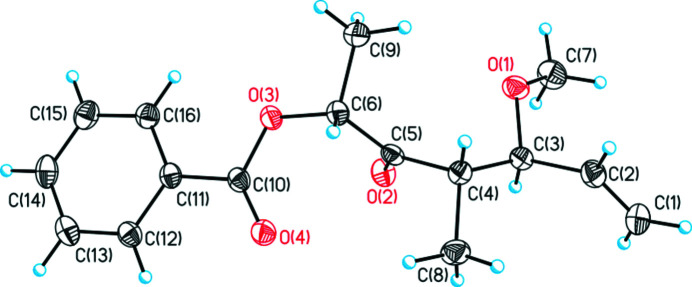
The mol­ecular structure of **I** showing displacement ellipsoids at the 50% probability level

**Figure 2:**
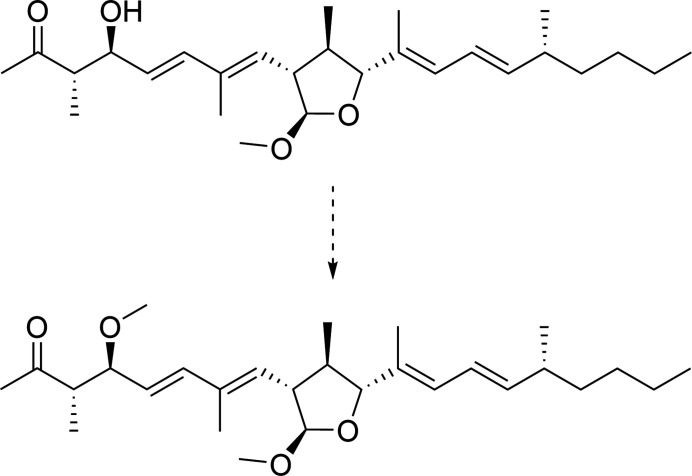
Methyl­ation of *O*-desmethyl­fusaequisin A.

The title compound, C_16_H_20_O_4_ (Fig. 1 ▸), was obtained during the synthesis of the Western fragment of fusaequisin A. Background to fusaequisin A is given by Shiono *et al.* (2013 ▸). The asymmetric synthesis of the Western fragment is based on Paterson’s *anti* aldol chemistry (Paterson *et al.*, 1994 ▸; Paterson, 1998 ▸). In the course of the total synthesis of curvicollide C (Che *et al.*, 2004 ▸) the precursor of the title compound (**I**) was prepared (von Kiedrowski *et al.*, 2017 ▸) and provided potential for further investigations regarding the total synthesis of fusaequisin A. The methyl­ation process is shown in Fig. 2 ▸.

The title compound crystallizes in the ortho­rhom­bic space group *P*2_1_2_1_2_1_ with four mol­ecules in the unit cell with H1*A* and H3*A* almost in plane (H1*A*—C1⋯C3—H3*A* pseudo torsion angle = −1°) and H2*A* and H3*A* in an anti­periplanar arrangement (H2*A*—C2—C3—H3*A* = 179°), which minimizes 1,3-allylic strain. Furthermore, the C8 methyl group and the O1 atom of the ether group are also in an anti­periplanar arrangement with a C8—C4—C3—O1 torsion angle of 177.32 (10)°. The ester moiety shows the most stable and expected *s–cis-*conformation. In the crystal, a weak C—H⋯O inter­action arising from the aromatic C—H grouping *para* to the side chain links the mol­ecules into *C*(10) chains propagating in the [010] direction (Table 1 ▸).

## Synthesis and crystallization

**Figure 3:**
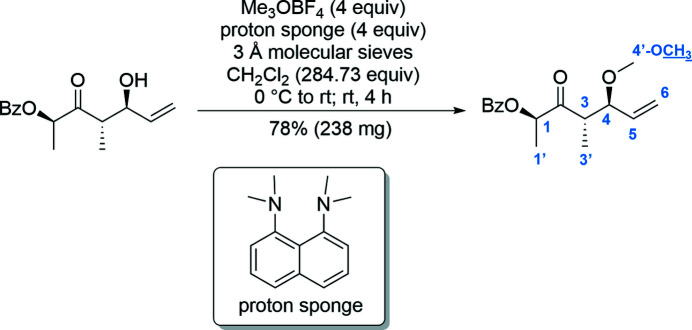
Reaction conditions for the methyl­ation of the allylic alcohol.

The reaction (Fig. 3 ▸) was carried out under an argon atmosphere. To an ice-cooled solution of the allylic alcohol (C_15_H_18_O_4_, 262.31 g mol^−1^, 300 mg, 1.10 mmol, 1 equiv.) in CH_2_Cl_2_ were successively added dried (0.1 mbar, 250°C, 2 h) 3 Å mol­ecular sieves (200 mg), 1,8-bis­(di­methyl­amino)­naphthalene (proton sponge^®^, C_14_H_18_N_2_, 214.31 g mol^−1^, 943 mg, 4.40 mmol, 4 equiv.) and tri­methyl­oxonium tetra­fluoro­borate (Me_3_OBF_4_, C_3_H_9_BF_4_O, 147.91 g mol^−1^, 651 mg, 4.40 mmol, 4 equiv.). The opaque, orange solution was warmed to room temperature. The reaction mixture was stirred at room temperature for 4 h and was then diluted by the addition of aqueous phosphate pH 7 buffer. The phases were separated and the aqueous layer was extracted three times with CH_2_Cl_2_. The combined organic layers were dried (MgSO_4_) and all volatiles were removed under reduced pressure. The light yellow residue was purified by flash chromatography (cyclo­hexane-ethyl acetate, 20:1 to 10:1) to afford the title methyl ether (**I**) (C_16_H_20_O_4_, 276.33 g mol^−1^, 238 mg, 0.86 mmol, 78%) as a white solid. Colourless crystals of **I** suitable for X-ray crystallographic analysis were obtained under air by slow evaporation from the mixed solvents of diethyl ether and *n*-pentane. *R*_f_ = 0.56 (cyclo­hexa­ne–ethyl acetate, 5:1); m.p. = 80–83°C; **[** ***a*** **]_D_^20^** = −8.3° (*c* = 0.5 g ml^−1^ in CHCl_3_) ; **^1^H NMR** (500 MHz, CDCl_3_) δ 1.06 (*d*, *J* = 7.1 Hz, 3H, 3-CH_3_), 1.55 (*d*, *J* = 7.0 Hz, 3H, 1-CH_3_), 2.93 (*dq*, *J* = 9.7, 7.1 Hz, 1H, 3-CH), 3.15 (*s*, 3H, 4-OCH_3_), 3.70 (*dd*, *J* = 10.1, 9.3 Hz, 1H, 4-CH), 5.24–5.35 (*m*, 2H, 6-CH_2_), 5.41 (*q*, *J* = 7.0 Hz, 1H, 1-CH), 5.56 (*ddd*, *J* = 17.1, 10.1, 8.5 Hz, 1H, 5-CH), 7.43–7.48 (*m*, 2H, aryl-CH), 7.55–7.60 (*m*, 1H, aryl-CH), 8.05–8.12 (*m*, 2H, aryl-CH); **^13^C NMR** (126 MHz, CDCl_3_) δ 14.1 (3-CH_3_), 15.3 (1-CH_3_), 47.0 (3-CH), 56.6 (4-OCH_3_), 75.5 (1-CH), 85.4 (4-CH), 120.2 (6-CH_2_), 128.5 (aryl-CH), 129.8 (aryl-CH), 129.9 (aryl-CH), 133.3 (aryl-CH), 136.0 (5-CH), 166.0 (aryl-C), 210.1 (2-C); **IR** ν = 3075 (*w*), 2985 (*w*), 2935 (*w*), 2825 (*w*), 1720 (*s*), 1065 (*w*), 1450 (*m*), 1420 (*w*), 1375 (*m*), 1315 (*m*), 1265 (*s*), 1205 (*w*), 1175 (*w*), 1115 (*s*), 1090 (*s*), 1070 (*m*), 1025 (*m*), 1010 (*m*), 965 (*m*), 935 (*m*), 715 (*s*), 685 (*w*) cm^−1^; **HRMS (ESI)**: *m*/*z* [*M* + H]^+^ calculated for C_16_H_21_O_4_: 277.1434; found: 277.1342.

## Refinement

Crystal data, data collection and structure refinement details are summarized in Table 2 ▸.

## Supplementary Material

Crystal structure: contains datablock(s) I. DOI: 10.1107/S2414314621009512/hb4390sup1.cif

Structure factors: contains datablock(s) I. DOI: 10.1107/S2414314621009512/hb4390Isup2.hkl

Supporting information file. DOI: 10.1107/S2414314621009512/hb4390Isup3.cdx

Supporting information file. DOI: 10.1107/S2414314621009512/hb4390Isup4.cml

CCDC reference: 2109383

Additional supporting information:  crystallographic information; 3D view; checkCIF report

## Figures and Tables

**Table 1:** Hydrogen-bond geometry (Å, °)

*D*—H⋯*A*	*D*—H	H⋯*A*	*D*⋯*A*	*D*—H⋯*A*
C14—H14*A*⋯O2^i^	0.95	2.54	3.2838 (18)	135

Symmetry code: (i) 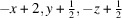 .

**Table 2:** Experimental details

Crystal data	
Chemical formula	C_16_H_20_O_4_
*M*_r_	276.32
Crystal system, space group	Orthorhombic, *P*2_1_2_1_2_1_
Temperature (K)	100
*a*, *b*, *c* (Å)	8.1297 (4), 11.8232 (6), 15.7213 (9)
*V* (Å^3^)	1511.12 (14)
*Z*	4
Radiation type	Cu *K*α
μ (mm^−1^)	0.71
Crystal size (mm)	0.12 × 0.10 × 0.06

Data collection	
Diffractometer	Bruker APEXII CCD
Absorption correction	Multi-scan (*SADABS*;Bruker, 2016 ▸)
*T*_min_, *T*_max_	0.700, 0.754
No. of measured, independent and observed [*I* > 2σ(*I*)] reflections	28627, 3078, 3054
*R*_int_	0.027
(sin θ/λ)_max_ (Å^−1^)	0.625

Refinement	
*R*[*F* ^2^ > 2σ(*F* ^2^)], *wR*(*F* ^2^), *S*	0.023, 0.060, 1.07
No. of reflections	3078
No. of parameters	184
H-atom treatment	H-atom parameters constrained
Δρ_max_, Δρ_min_ (e Å^−3^)	0.18, −0.12
Absolute structure	Flack *x* determined using 1293 quotients [(*I* ^+^)−(*I* ^−^)]/[(*I* ^+^)+(*I* ^−^)] (Parsons *et al.*, 2013 ▸)
Absolute structure parameter	0.03 (2)

Computer programs: *APEX3* and *SAINT* (Bruker, 2016 ▸), *SHELXT2014/5* (Sheldrick, 2015*a* ▸), *SHELXL2018/3* (Sheldrick, 2015*b* ▸), *XP* (Sheldrick, 2008 ▸) and *OLEX2* (Dolomanov *et al.*, 2009 ▸).

## full crystallographic data

### Crystal data

**Table:**

C_16_H_20_O_4_	*D*_x_ = 1.215 Mg m^−^^3^
*M**_r_* = 276.32	Melting point = 353–356 K
Orthorhombic, *P*2_1_2_1_2_1_	Cu *K*α radiation, λ = 1.54178 Å
*a* = 8.1297 (4) Å	Cell parameters from 9807 reflections
*b* = 11.8232 (6) Å	θ = 6.1–74.6°
*c* = 15.7213 (9) Å	µ = 0.71 mm^−^^1^
*V* = 1511.12 (14) Å^3^	*T* = 100 K
*Z* = 4	Block, colourless
*F*(000) = 592	0.12 × 0.10 × 0.06 mm

### Data collection

**Table:**

Bruker APEXII CCD diffractometer	3054 reflections with *I* > 2σ(*I*)
φ and ω scans	*R*_int_ = 0.027
Absorption correction: multi-scan (SADABS;Bruker, 2016)	θ_max_ = 74.5°, θ_min_ = 4.7°
*T*_min_ = 0.700, *T*_max_ = 0.754	*h* = −10→10
28627 measured reflections	*k* = −14→14
3078 independent reflections	*l* = −18→19

### Refinement

**Table:**

Refinement on *F*^2^	Hydrogen site location: inferred from neighbouring sites
Least-squares matrix: full	H-atom parameters constrained
*R*[*F*^2^ > 2σ(*F*^2^)] = 0.023	*w* = 1/[σ^2^(*F*_o_^2^) + (0.0308*P*)^2^ + 0.2127*P*] where *P* = (*F*_o_^2^ + 2*F*_c_^2^)/3
*wR*(*F*^2^) = 0.060	(Δ/σ)_max_ < 0.001
*S* = 1.07	Δρ_max_ = 0.18 e Å^−^^3^
3078 reflections	Δρ_min_ = −0.12 e Å^−^^3^
184 parameters	Absolute structure: Flack *x* determined using 1293 quotients [(*I*^+^)-(*I*^-^)]/[(*I*^+^)+(*I*^-^)] (Parsons *et al.*, 2013)
0 restraints	Absolute structure parameter: 0.03 (2)
Primary atom site location: dual	

### Special details

**Table:**

Geometry. All esds (except the esd in the dihedral angle between two l.s. planes) are estimated using the full covariance matrix. The cell esds are taken into account individually in the estimation of esds in distances, angles and torsion angles; correlations between esds in cell parameters are only used when they are defined by crystal symmetry. An approximate (isotropic) treatment of cell esds is used for estimating esds involving l.s. planes.

### Fractional atomic coordinates and isotropic or equivalent isotropic displacement parameters (Å^2^)

**Table:**

	*x*	*y*	*z*	*U*_iso_*/*U*_eq_	
C1	0.09507 (18)	0.29265 (12)	0.39623 (10)	0.0306 (3)	
H1A	0.080921	0.309404	0.337584	0.037*	
H1B	0.010147	0.255322	0.426807	0.037*	
O1	0.52090 (11)	0.31350 (8)	0.40027 (6)	0.0257 (2)	
C2	0.23199 (17)	0.32109 (12)	0.43502 (9)	0.0260 (3)	
H2A	0.241816	0.302998	0.493680	0.031*	
O2	0.57315 (12)	0.55222 (8)	0.31396 (6)	0.0257 (2)	
C3	0.37402 (15)	0.38013 (10)	0.39331 (8)	0.0210 (3)	
H3A	0.348192	0.392678	0.331840	0.025*	
O3	0.79670 (11)	0.67523 (7)	0.40634 (6)	0.02254 (19)	
C4	0.41493 (15)	0.49330 (10)	0.43492 (8)	0.0200 (2)	
H4A	0.442788	0.480340	0.496105	0.024*	
O4	0.58296 (11)	0.79534 (7)	0.39300 (6)	0.0255 (2)	
C5	0.56184 (15)	0.54690 (10)	0.39052 (8)	0.0195 (2)	
C6	0.69017 (16)	0.59668 (11)	0.45003 (8)	0.0214 (3)	
H6A	0.633436	0.636762	0.497741	0.026*	
C7	0.5209 (2)	0.22018 (12)	0.34321 (10)	0.0346 (3)	
H7A	0.624899	0.178862	0.348492	0.052*	
H7B	0.508591	0.247754	0.284770	0.052*	
H7C	0.429206	0.169617	0.357046	0.052*	
C8	0.27180 (16)	0.57771 (12)	0.42921 (10)	0.0279 (3)	
H8A	0.180721	0.551481	0.464968	0.042*	
H8B	0.234674	0.583387	0.370058	0.042*	
H8C	0.308649	0.652131	0.448962	0.042*	
C9	0.80093 (17)	0.50522 (12)	0.48637 (10)	0.0298 (3)	
H9A	0.733683	0.448423	0.515567	0.045*	
H9B	0.878182	0.539103	0.526875	0.045*	
H9C	0.862385	0.469110	0.440132	0.045*	
C10	0.72720 (16)	0.77408 (10)	0.38331 (7)	0.0204 (3)	
C11	0.85150 (16)	0.85390 (10)	0.34781 (8)	0.0206 (3)	
C12	0.79937 (18)	0.96224 (11)	0.32518 (8)	0.0237 (3)	
H12A	0.686828	0.982661	0.330513	0.028*	
C13	0.91308 (19)	1.04000 (11)	0.29482 (9)	0.0286 (3)	
H13A	0.878073	1.114116	0.280093	0.034*	
C14	1.07716 (19)	1.01054 (12)	0.28578 (9)	0.0303 (3)	
H14A	1.153904	1.064064	0.264420	0.036*	
C15	1.12937 (17)	0.90223 (13)	0.30810 (9)	0.0294 (3)	
H15A	1.241701	0.881748	0.301761	0.035*	
C16	1.01703 (17)	0.82427 (11)	0.33962 (8)	0.0243 (3)	
H16A	1.052798	0.750788	0.355613	0.029*	

### Atomic displacement parameters (Å^2^)

**Table:**

	*U*^11^	*U*^22^	*U*^33^	*U*^12^	*U*^13^	*U*^23^
C1	0.0290 (7)	0.0303 (7)	0.0325 (7)	−0.0065 (6)	0.0030 (6)	0.0020 (6)
O1	0.0265 (4)	0.0206 (4)	0.0298 (5)	0.0050 (4)	−0.0010 (4)	−0.0022 (4)
C2	0.0284 (7)	0.0248 (6)	0.0249 (6)	−0.0012 (5)	0.0023 (5)	0.0032 (5)
O2	0.0317 (5)	0.0244 (4)	0.0211 (4)	−0.0027 (4)	0.0034 (4)	−0.0001 (3)
C3	0.0220 (6)	0.0199 (6)	0.0210 (6)	0.0009 (5)	0.0000 (5)	0.0011 (5)
O3	0.0210 (4)	0.0178 (4)	0.0289 (5)	−0.0008 (4)	0.0021 (4)	0.0021 (4)
C4	0.0197 (5)	0.0207 (6)	0.0197 (5)	0.0004 (5)	0.0012 (5)	−0.0017 (5)
O4	0.0226 (4)	0.0211 (4)	0.0326 (5)	0.0007 (4)	0.0033 (4)	−0.0017 (4)
C5	0.0215 (6)	0.0146 (5)	0.0224 (6)	0.0040 (5)	0.0012 (5)	0.0001 (5)
C6	0.0210 (6)	0.0199 (6)	0.0234 (6)	−0.0018 (5)	0.0013 (5)	0.0028 (5)
C7	0.0377 (8)	0.0236 (6)	0.0426 (8)	0.0051 (6)	0.0039 (7)	−0.0090 (6)
C8	0.0234 (6)	0.0264 (7)	0.0339 (7)	0.0053 (5)	0.0006 (6)	−0.0059 (5)
C9	0.0233 (6)	0.0279 (7)	0.0382 (7)	−0.0005 (6)	−0.0036 (6)	0.0095 (6)
C10	0.0244 (6)	0.0174 (5)	0.0196 (6)	−0.0006 (5)	−0.0008 (5)	−0.0030 (5)
C11	0.0245 (6)	0.0192 (6)	0.0180 (6)	−0.0020 (5)	0.0002 (5)	−0.0034 (5)
C12	0.0277 (7)	0.0209 (6)	0.0226 (6)	0.0005 (5)	0.0004 (5)	−0.0015 (5)
C13	0.0389 (7)	0.0214 (6)	0.0256 (6)	−0.0034 (6)	0.0022 (6)	0.0009 (5)
C14	0.0354 (7)	0.0296 (7)	0.0260 (6)	−0.0119 (6)	0.0064 (6)	−0.0015 (5)
C15	0.0252 (7)	0.0340 (7)	0.0291 (7)	−0.0038 (6)	0.0038 (5)	−0.0049 (6)
C16	0.0257 (6)	0.0236 (6)	0.0235 (6)	−0.0003 (5)	0.0003 (5)	−0.0026 (5)

### Geometric parameters (Å, º)

**Table:**

C1—C2	1.313 (2)	C7—H7B	0.9800
C1—H1A	0.9500	C7—H7C	0.9800
C1—H1B	0.9500	C8—H8A	0.9800
O1—C7	1.4220 (17)	C8—H8B	0.9800
O1—C3	1.4347 (15)	C8—H8C	0.9800
C2—C3	1.5001 (18)	C9—H9A	0.9800
C2—H2A	0.9500	C9—H9B	0.9800
O2—C5	1.2087 (16)	C9—H9C	0.9800
C3—C4	1.5261 (17)	C10—C11	1.4911 (18)
C3—H3A	1.0000	C11—C12	1.3953 (18)
O3—C10	1.3477 (15)	C11—C16	1.3965 (19)
O3—C6	1.4438 (15)	C12—C13	1.3883 (19)
C4—C5	1.5215 (17)	C12—H12A	0.9500
C4—C8	1.5357 (17)	C13—C14	1.386 (2)
C4—H4A	1.0000	C13—H13A	0.9500
O4—C10	1.2089 (16)	C14—C15	1.394 (2)
C5—C6	1.5199 (17)	C14—H14A	0.9500
C6—C9	1.5188 (18)	C15—C16	1.389 (2)
C6—H6A	1.0000	C15—H15A	0.9500
C7—H7A	0.9800	C16—H16A	0.9500
			
C2—C1—H1A	120.0	H7B—C7—H7C	109.5
C2—C1—H1B	120.0	C4—C8—H8A	109.5
H1A—C1—H1B	120.0	C4—C8—H8B	109.5
C7—O1—C3	112.20 (11)	H8A—C8—H8B	109.5
C1—C2—C3	124.65 (12)	C4—C8—H8C	109.5
C1—C2—H2A	117.7	H8A—C8—H8C	109.5
C3—C2—H2A	117.7	H8B—C8—H8C	109.5
O1—C3—C2	110.60 (10)	C6—C9—H9A	109.5
O1—C3—C4	105.50 (10)	C6—C9—H9B	109.5
C2—C3—C4	112.85 (10)	H9A—C9—H9B	109.5
O1—C3—H3A	109.3	C6—C9—H9C	109.5
C2—C3—H3A	109.3	H9A—C9—H9C	109.5
C4—C3—H3A	109.3	H9B—C9—H9C	109.5
C10—O3—C6	115.73 (10)	O4—C10—O3	123.58 (12)
C5—C4—C3	109.85 (10)	O4—C10—C11	124.96 (12)
C5—C4—C8	107.29 (10)	O3—C10—C11	111.42 (11)
C3—C4—C8	112.31 (10)	C12—C11—C16	119.97 (12)
C5—C4—H4A	109.1	C12—C11—C10	118.07 (12)
C3—C4—H4A	109.1	C16—C11—C10	121.92 (12)
C8—C4—H4A	109.1	C13—C12—C11	119.56 (13)
O2—C5—C6	122.72 (12)	C13—C12—H12A	120.2
O2—C5—C4	122.56 (12)	C11—C12—H12A	120.2
C6—C5—C4	114.69 (10)	C14—C13—C12	120.64 (13)
O3—C6—C9	106.34 (10)	C14—C13—H13A	119.7
O3—C6—C5	111.60 (10)	C12—C13—H13A	119.7
C9—C6—C5	111.27 (11)	C13—C14—C15	119.88 (13)
O3—C6—H6A	109.2	C13—C14—H14A	120.1
C9—C6—H6A	109.2	C15—C14—H14A	120.1
C5—C6—H6A	109.2	C16—C15—C14	119.95 (13)
O1—C7—H7A	109.5	C16—C15—H15A	120.0
O1—C7—H7B	109.5	C14—C15—H15A	120.0
H7A—C7—H7B	109.5	C15—C16—C11	120.00 (13)
O1—C7—H7C	109.5	C15—C16—H16A	120.0
H7A—C7—H7C	109.5	C11—C16—H16A	120.0
			
C7—O1—C3—C2	75.42 (14)	O2—C5—C6—C9	−102.56 (14)
C7—O1—C3—C4	−162.25 (11)	C4—C5—C6—C9	79.43 (13)
C1—C2—C3—O1	−121.33 (15)	C6—O3—C10—O4	−4.44 (17)
C1—C2—C3—C4	120.75 (15)	C6—O3—C10—C11	173.50 (10)
O1—C3—C4—C5	58.00 (12)	O4—C10—C11—C12	1.17 (19)
C2—C3—C4—C5	178.86 (10)	O3—C10—C11—C12	−176.74 (11)
O1—C3—C4—C8	177.32 (10)	O4—C10—C11—C16	178.96 (13)
C2—C3—C4—C8	−61.81 (14)	O3—C10—C11—C16	1.06 (16)
C3—C4—C5—O2	46.33 (16)	C16—C11—C12—C13	−0.16 (19)
C8—C4—C5—O2	−76.02 (15)	C10—C11—C12—C13	177.67 (11)
C3—C4—C5—C6	−135.66 (10)	C11—C12—C13—C14	0.8 (2)
C8—C4—C5—C6	101.99 (12)	C12—C13—C14—C15	−0.6 (2)
C10—O3—C6—C9	−167.80 (11)	C13—C14—C15—C16	−0.2 (2)
C10—O3—C6—C5	70.68 (13)	C14—C15—C16—C11	0.9 (2)
O2—C5—C6—O3	16.05 (17)	C12—C11—C16—C15	−0.70 (19)
C4—C5—C6—O3	−161.96 (10)	C10—C11—C16—C15	−178.45 (12)

### Hydrogen-bond geometry (Å, º)

**Table:**

*D*—H···*A*	*D*—H	H···*A*	*D*···*A*	*D*—H···*A*
C14—H14*A*···O2^i^	0.95	2.54	3.2838 (18)	135

Symmetry code: (i) −*x*+2, *y*+1/2, −*z*+1/2.

## References

[bb1] Bruker (2016). *APEX3*, *SAINT* and *SADABS* (version 2016/2). Bruker AXS Inc., Madison, Wisconsin, USA.

[bb2] Che, Y., Gloer, J. B. & Wicklow, D. T. (2004). *Org. Lett.* **6**, 1249–1252. 10.1021/ol0498186 15070309

[bb3] Dolomanov, O. V., Bourhis, L. J., Gildea, R. J., Howard, J. A. K. & Puschmann, H. (2009). *J. Appl. Cryst.* **42**, 339–341.

[bb4] Kiedrowski, V. von, Quentin, F. & Hiersemann, M. (2017). *Org. Lett.* **19**, 4391–4394. 10.1021/acs.orglett.7b02126 28763235

[bb5] Parsons, S., Flack, H. D. & Wagner, T. (2013). *Acta Cryst.* B**69**, 249–259. 10.1107/S2052519213010014 PMC3661305 23719469

[bb6] Paterson, I. (1998). *Synthesis*, pp. 639–652.

[bb7] Paterson, I., Wallace, D. J. & Velázquez, S. M. (1994). *Tetrahedron Lett.* **35**, 9083–9086.

[bb8] Sheldrick, G. M. (2008). *Acta Cryst.* A**64**, 112–122. 10.1107/S0108767307043930 18156677

[bb9] Sheldrick, G. M. (2015*a*). *Acta Cryst.* A**71**, 3–8.

[bb10] Sheldrick, G. M. (2015*b*). *Acta Cryst.* C**71**, 3–8.

[bb11] Shiono, Y., Shibuya, F., Murayama, T., Koseki, T., Poumale, H. M. P. & Ngadjui, B. T. (2013). *Z. Naturfosch. Teil B*, **68**, 289–292.

